# Relationship Context Moderates Couple Congruence in Ratings of Sexual Arousal and Pain During Vaginal Sensations in the Laboratory

**DOI:** 10.1007/s10508-019-1452-3

**Published:** 2019-09-03

**Authors:** Marieke Dewitte, Jan Schepers

**Affiliations:** 1 Department of Clinical Psychological Science, Maastricht University, Universiteitssingel, 40, 6229 ER Maastricht, The Netherlands; 2 Department of Methodology and Statistics, Maastricht University, Maastricht, The Netherlands

**Keywords:** Genital pain, Sexual arousal, Relationship satisfaction, Perceived partner responsiveness, Interpersonal relations, DSM-5

## Abstract

Genital pain is a social experience that needs to be studied as a dyadic interaction between partners. The present study relied on a sample of 42 heterosexual couples to examine the level of congruence between both partners’ ratings of pain and sexual arousal in response to experimentally induced vaginal pressure that served as a simulation of vaginal sensations during penetration. We also inferred the men’s ability to estimate their partner’s level of pain and sexual arousal. Because the relationship has shown to influence pain estimations, we considered the moderating role of perceived partner responsiveness and relationship satisfaction. We found higher disagreement in pain ratings when vaginal pressure was induced in the context of a sexual film compared to a neutral film, with men overestimating the level of pain in women. Also sexual arousal ratings diverged between partners, with men underestimating their partners’ level of sexual arousal during the induction of vaginal pressure, regardless of whether they were watching a sexual or neutral film. Importantly, the level of congruence between actual and estimated ratings of pain and sexual arousal depended on how relationally satisfied men and women were and how validated and supported women felt by their male partner. These results make an important contribution to the growing literature on the social determinants of sexual pain experiences.

## Introduction

Although female genital pain^1^ is a personal experience, it typically occurs within an interpersonal context, with the partner both triggering and witnessing the pain of the woman (Bergeron, Rosen, & Morin, 2011; Dewitte, van Lankveld, & Crombez, 2011; Rosen, Rancourt, Corsini-Munt, & Bergeron, 2014). Accordingly, interpersonal accounts of genital pain are gaining ground, describing pain as a social experience that serves interpersonal dynamics and thus needs to be studied as a dyadic interaction between partners (Dewitte 2014). This shift from focusing on the individual toward studying how the partner and the relationship context affect and are affected by the woman’s pain has been witnessed in both research and clinical practice and continues to develop. The present study adds to this line of research by using an experimental laboratory design to study the level of congruence between both partners’ ratings of pain and sexual arousal. We thereby focus particularly on the role of the male partner’s estimations of pain and sexual arousal.

### The Role of the Partner in Chronic and Genital Pain

Within the chronic pain literature, several models have been proposed to explain how the partner modulates the patient’s pain experience. The communications model of pain, for example, describes how the woman in pain may (in)voluntary communicate pain and associated distress with her partner, who will make inferences about the pain experience of the woman and display emotional and behavioral responses, which will, in turn, affect the woman’s experience and expression of pain (Hadjistavropoulos et al., 2011). In essence, this model coincides with an operant framework in which partner responses are mainly approached as positive or negative reinforcers of pain (Fordyce, 1976). Special attention has been paid to identifying and changing solicitous support because this is rewarding to the woman in pain and positively reinforces displays of pain behavior, which eventually prolongs the pain (Cano, 2013; Rosen, Bergeron, Glowacka, Delisle, & Baxter, 2012). Hostile responses, on the other hand, discourage expressions of pain, but have a negative effect on the couple’s well-being. Most adaptive are facilitative responses in which the partner encourages the woman to engage in non-painful sexual activities (Rosen et al., 2012). Although this operant view continues to influence the pain literature and clinical interventions, its unidirectional, instrumental focus has been criticized because it falls short in capturing the nuances and complexity of the interpersonal dynamics of pain (Bergeron et al., 2011; Cano & Williams, 2010). Moving beyond the individual implies taking into account the dyadic interaction between partners instead of studying only the impact of partner responses on the pain experience because then the focus remains on the woman in pain.

Recently, intimacy-based models have been proposed that take the couple as unit of analysis and describe the impact of pain on intimacy and emotion regulation in couples (Cano & Williams, 2010). According to this view, partner responses may serve to enhance one’s need for intimacy. A supportive partner can exert positive effects and empower the woman in pain when his support matches their need for intimacy and closeness (Edmond & Keefe, 2015). Accordingly, intimacy will develop when a person’s disclosure of pain is met with empathic and validating responses of the partner (Goubert et al., 2005; Goubert, Vervoort, & Craig, 2013; Laurenceau, Barrett, & Pietromonaco, 1998; Mitchell et al., 2008; Reis, Clark, & Holmes, 2004; Reis & Shaver, 1988). Verifying this, research has shown that higher levels of intimacy, defined in terms of empathy and disclosure, are associated with a better adaptation to pain because intimate interactions between partners decrease sexual distress and increase sexual and relationship satisfaction (Bois, Bergeron, Rosen, McDuff, & Grégoire, 2013; Rosen, Bois, Mayrand, Vannier, & Bergeron, 2016). A crucial aspect of intimacy is the partner’s capacity to empathize with the other. Labeled as perceived partner responsiveness (Reis et al., 2004), it has been shown that expressions of validation, affection, and investment in the relationship contribute to greater relationship well-being and better coping with pain because this reinforces the perception that the couple is facing the pain problem together (Dagan et al., 2014; Rosenbaum, Barnard, & Wilhite, 2015).

### The Partners’ Estimations of Pain and Sexual Arousal

In the context of chronic and genital pain, an important determinant of empathy and partner responsiveness is the partner’s ability to estimate the woman’s pain because this allows him to adjust his emotional and behavioral responses. This will eventually contribute to more adaptive coping and higher satisfaction with the (sexual) relationship (Rosen, Sadikaj, & Bergeron, 2015). Although the partner may be generally motivated to accurately perceive and attune to the woman’s pain, it is also plausible to assume that he will defensively refrain from observing the full extent to which the pain problem disables the woman. Previous research on chronic pain has shown that women’s actual and partners’ estimated pain reports covary to a certain degree, which suggests that the partner is relatively accurate in estimating the woman’s pain (Cano, Johansen, & Franz, 2005; Gauthier, Thibault, & Sullivan, 2008; Leonard, Issner, Cano, & Williams, 2013). Specific to genital pain, a daily diary study has shown that the partner is able to accurately track changes in the woman’s pain, although they do, in general, underestimate her pain (Rosen et al., 2015). This study has also demonstrated that the accuracy of pain estimations is contingent on the relationship context. Low commitment and poor relationship satisfaction may undermine the partner’s motivation to understand the woman’s pain or interfere with the woman’s ability to openly communicate about her pain experiences (Lyons, Jones, Bennet, Hiatt, & Sayer, 2013).

Adaptive coping to pain depends not only on the partner’s perception of pain, it is also important that men can accurately estimate how sexually aroused the woman is because high sexual arousal is a prerequisite for having painless sex (Spano & Lamont, 1975, ter Kuile, Both, & van Lankveld, 2010). This implies that men should stop initiating penetrative sex at the moment they perceive that their partner is not sexually aroused yet. Previous work has shown that, in the context of initial encounters and short-term relationships, men display an overperception bias wherein they systematically perceive more sexual interest in a woman’s behavior than actually exists (Haselton & Buss, 2000; Henningsen & Henningsen, 2010). In more established relationships, however, men are likely to underperceive their partner’s desire, especially on days when they are motivated to avoid sexual rejection, which eventually functions to safeguard a satisfying relationship climate (Muise, Stanton, Kim, & Impett, 2016). This suggests that the relationship context plays an important role in modulating men’s perceptions of sexual interest. In the context of genital pain, no research—to the best of our knowledge—has examined whether men are able to estimate the woman’s level of sexual arousal when faced with a potentially painful sexual encounter and whether their judgment is biased by relationship processes.

Based on previous work, we can thus conclude that understanding couple dynamics in response to genital pain requires focusing not only on the partner’s actual responses to the pain, but to examine perceptional biases of pain and sexual arousal. These estimates will eventually regulate the partner’s emotional and behavioral responses to pain. It is plausible to assume that not the partner’s perception or responses per se, but rather the mismatch between actual and perceived pain responses will explain maladaptive coping and feelings of dissatisfaction (Cano, Johansen, & Geisser, 2004). Although previous studies have already revealed valuable information on this behalf, using a range of methods that include retrospective surveys (Bois et al., 2013) and prospective daily diaries (Rosen et al., 2015), more research is needed to gain deeper insight into the dyadic interplay between the partners’ actual and perceived pain and sexual arousal. To this aim, research might benefit from experimental studies in which responses are measured in a sexually stimulating context; in real time, thus at the moment of potential pain induction; and in response to an experimental pain stimulus that is induced in a systematic way, thereby allowing more experimental control over the processes involved in dyadic genital pain responding. Testing this in a laboratory context allows maximum internal validity as well as a process-pure analysis of the impact of vaginal sensations on sexual arousal and pain ratings.

### The Present Study

In the present study, we used an experimental laboratory design to examine the level of agreement between women’s actual and men’s estimated reports of pain and sexual arousal in order to evaluate the accuracy of partner’s estimations when the woman is confronted with a potentially painful sexual stimulus. Using a recently developed device, the Vaginal Pressure Inducer (VPI), we induced prolonged and dispersed vaginal pressure in the outer third part of the vagina, exactly there where the penis exerts the largest pressure during penetration (Melles, Dewitte, ter Kuile, Bonnemayer, & Peters, 2017). As such, the VPI simulates the vaginal sensations that women experience during penetrative sex. Although this pressure sensation does not reflect a discrete pain stimulus, we believe the VPI is well suited to study the underlying dynamics of penetration pain because women—particularly those suffering from provoked vulvodynia—seem to appraise vaginal pressure at initial penetration as most painful (Farmer et al., 2013). Previous research with the VPI has shown that the experience of vaginal pressure depends on the level of sexual stimulation, with higher levels of sexual arousal leading to an increase in pleasurable sensitivity and unpleasantness thresholds (Melles et al., 2017). We relied on a within-subjects design in which women watched a sexual versus neutral movie, while vaginal pressure was induced or not, in the presence versus absence of the partner. The effects of partner presence on sexual arousal and pain ratings are reported elsewhere (Dewitte et al., 2018b). In this study, we consider only the partner-present condition so that, during the pressure induction, men were able to observe their partner. This implies that they had visual cues (e.g., facial expressions and vocalizations) to rely on when making their judgment instead of providing estimates based on previous experiences or personality features of the women.

#### Hypotheses

Assuming that perfect agreement between partners is improbable (Kappesser & Williams, 2010), we expected that men will underestimate the women’s pain during a potentially painful vaginal experience, both in a sexual and non-sexual context. Based on previous studies, we also hypothesized that men will underestimate the women’s level of sexual arousal, especially in response to potentially painful vaginal pressure. Because previous work has ascribed an important role to relational intimacy in determining perceptional biases and couple congruence, we considered the moderating role of relationship satisfaction and perceived partner responsiveness, with the latter being a crucial determinant of relationship intimacy. Given the protective role of intimacy in the face of adversity (Cano & Williams, 2010; Laurenceau et al., 1998), we expected that perceptual biases (and thus the level of agreement between partners) will be smaller in the context of a satisfying relationship in which couple members feel understood and validated by a partner who is perceived as available and responsive. Because there are no studies available yet to build these hypotheses on, we did not make specific predictions on whether it is either the men’s, the women’s, or their both reports of perceived partner responsiveness and relationship satisfaction that will moderate the level of agreement between couple members. For exploratory reasons, we also examined whether men’s estimations of pain and sexual arousal in women will relate to their own level of sexual arousal. We expected that men will report less sexual arousal when they perceive more pain in their partner, whereas higher estimates of sexual arousal are assumed to fuel their own level of sexual arousal.

## Method

### Participants

Participants were recruited via advertisements at the university, Facebook, and personal contacts of the research assistant involved in the study. Participants who were interested in a study on “couple dynamics in sexual arousal” could freely sign up via an online subscription system. In total, 51 couples signed up for the experiment, of which four couples were excluded because they did not fit the criteria and five couples dropped out because they were no longer interested after reading the information letter in which the vaginal pressure instrument was explained. Eventually, 42 couples participated in this study. For both partners, inclusion criteria included the absence of sexual problems, aged between 18 and 45 years, good command of the Dutch language, a steady heterosexual relationship for at least 6 months, and being sexually active (defined as having coitus). Women were excluded in case of pregnancy, breastfeeding, (post-)menopause, major affective disorder, psychotic disorder, substance-related disorder, posttraumatic stress disorder, or if they were taking medication that likely interferes with sexuality. Women ranged in age from 18 to 44 years (*M* = 22, SD = 4.3), and men ranged in age from 18 to 45 years (*M* = 23.10, SD = 5.04). The average relationship length of the couples was 2.2 years (ranging from 6 months to 8 years). Both men and women were highly educated (69% higher education in women and 68% higher education in men). The majority of couples had no children, with only two couples having two children each. The mean score of the Female Sexual Functioning Index (FSFI; Rosen et al., 2000) was 31.04 (SD = 2.30) and men scored on average 68.64 (SD = 2.30) on the International Index of Erectile Function (IIEF; Rosen et al., 1997), indicating that sexual functioning was within a normal range for both men and women. The study was approved by the local Ethical Committee.

### Material

#### Stimulus Material

Two neutral and two sexual film clips of 5 min each were selected. The neutral film clips were two fragments of a documentary on nature (i.e., planet earth, BBC One). The sexual film clips depicted female-friendly porn scenes, displaying a combination of seductive acts, kissing, petting as well as manual, oral, and penetrative sex. Film clips were drawn from a popular porn site and selected based on the criteria recommended by Janssen and colleagues (Janssen, Carpenter, & Graham, 2003).

#### Vaginal Pressure Inducer (VPI)

The VPI consists of an inflatable vaginal balloon that is partially inserted into a synthetic handle. The handle ends in a flange, which can be placed against the opening of the vagina, thereby ensuring that the vaginal pressure is located at the introitus (for a more detailed description of the VPI, see Melles et al., 2017). The balloon is connected to a warm water tank that gradually pumps water (at body temperature) into the balloon until it reaches a length of 4–6 cm. When the balloon is filled, an outward omnidirectional pressure is given to the surrounding tissues. The participant can insert and adjust the VPI herself, and it can be remotely controlled to respect privacy of the participant. Previous research has indicated that the VPI is a valid instrument to investigate various determinants of unpleasant vaginal pressure (Melles et al., 2017) and that it is sensitive to context manipulations (Dewitte et al., 2018a, 2018b).

#### Subjective Measures

In each condition, both men and women reported on their subjective sexual arousal, “At this moment, to which extent do you feel sexually aroused?” Feeling sexually aroused was defined as how mentally sexually aroused the participants feel while watching the films. In addition, men were asked to provide an estimate of the level of sexual arousal of their female partner, “At this moment, to which extent does your partner feel sexually aroused?” Women also reported on their subjective experience of pleasant and painful vaginal pressure sensations, “While watching the film, to which extent did you experience a pleasant pressure/painful pressure in the vagina?” Likewise, men were asked to provide an estimate of the level of painful vaginal pressure in their female partner, “To which extent did your partner experience the vaginal pressure as painful?” All items were rated by placing a mark on a seven-point scale ranging from “not at all” to “very strong.”

#### Questionnaires

General relationship satisfaction was measured using the Maudsley Marital Questionnaire, containing 20 statements to which participants responded on nine-point Likert scales (e.g., When there is an argument, can you reach agreement? Can you tell your partner as much as you want?). Higher scores indicate greater relationship dissatisfaction. The MMQ scale has been used in both married and non-married couples (Hendrickx, Gijs, Janssen, & Enzlin, 2016) and has good psychometric properties (Arrindell, Boelens, & Lambert, 1983). This was supported by the current sample, in which reliability (Cronbach’s alpha) was good, *α* = .83 for women and *α* = .78 for men.

The Perceived Partner Responsiveness Scale (Reis, 2006; Reis et al., 2004) was used to assess how responsive and understanding the partner is perceived. Participants were asked to evaluate 10 items (e.g., “My partner sees the real me”) and rate them on seven-point Likert scales, ranging from one = *not at all true* to nine = *completely true*. Items were averaged so that higher scores indicate greater perceived partner responsiveness. In the current study, Cronbach’s alpha was *α *= .84 for women and *α* = .81 for men.

### Procedure

This study was part of a larger project in which we investigated the effect of partner presence on the sexual arousal and appraisal of vaginal pressure in women. In this protocol, men and women watched neutral and sexual film fragments, with and without vaginal pressure, one time while being together in the same room and one time while sitting apart in adjacent rooms. The results on the effects of partner presence on the appraisal of vaginal pressure and sexual arousal are reported elsewhere (Dewitte et al., 2018a, 2018b). For the present study, we focused only on the partner-present condition because we are primarily interested in testing whether men are able to interpret the sexual arousal and pain signals of their female partner in real time. Accordingly, we report only the procedural phases that are relevant for the present study. A full description of the experimental procedure can be found in the corresponding paper on the effects of partner presence.

The experiment took place in a sound-attenuated room at the university. Both male and female partners were fully informed about the procedure of the experiment before signing the informed consent. It was explained that they would watch a set of neutral and sexual film fragments and that they were not allowed to communicate or touch each other. During one of the two neutral and one of the two sexual film fragments, vaginal pressure was induced in the woman, which she could terminate by pressing a button that was placed on her lap. The woman was instructed to press the button as soon as the pressure felt unpleasant. Both partners were instigated to simply watch the film fragments and answer the questions. After having received the opportunity to ask further questions about the procedure of the experiment, the participants entered the laboratory rooms which were closed to guarantee complete privacy during the experiment. Any further communication occurred via the intercom.

At the start of the experiment, participants were administered a general questionnaire asking about demographic information. To acclimatize the woman, we first presented a neutral film with pressure induction. This condition was followed by a neutral film without pressure, a sex film with pressure, and a sex film without pressure. Because the neutral film conditions acted as a comparison base and we wanted to prevent carryover effects between the neutral and sex conditions, the order of the neutral films was kept constant (always presenting a neutral film with pressure first) while the order of the sex films with and without pressure was counterbalanced. To prevent carryover effects between the sex film with and without vaginal pressure, women completed a letter span working memory task of 5 min in between the films.

In all conditions with pressure induction, the vaginal balloon was gradually filled, 2 min after the start of the film, until the participant pressed a button. In the latter case, the movie stopped and the VPI balloon was immediately deflated to its initial level by draining the water. If the participant did not press the button, the movie ended after 5 min. At the end of each condition, the women completed questions on their sexual arousal and appraisal of vaginal pressure. The women watched the film fragments on a computer screen that was placed in front of them, and they responded to the questions via a wireless keyboard that was placed on their lap. The male partners were sitting opposite to the women (having a clear view on their partner and being able to observe the experimental procedure that induced vaginal pressure). They watched the same sequence of film fragments via a tablet that was placed on their lap. After each film, men reported on their own sexual arousal and provided an estimate of the level of sexual arousal and painful pressure of their female partner.

### Data Analyses

The level of (in) congruence between partners’ ratings of sexual arousal and painful vaginal pressure was investigated in two complementary ways: (1) by estimating the association between partners’ reports, and (2) by examining the mean difference between partners’ average ratings across couples.^2^ Accordingly, we first conducted a series of correlational analyses to examine the interrelation between women’s actual and men’s estimated reports of sexual arousal and painful pressure. Next, we examined the level of congruence between the average sexual arousal and painful pressure ratings as reported by women and estimated by men, as a function of pressure induction and sexual stimulation. For this purpose, we conducted a series of repeated measures ANOVA, entering gender (women’s responses vs. men’s estimations), film type (sex vs. neutral), and vaginal pressure (with vs. without) as within-subjects factors. In addition, we examined the moderating influence of relationship satisfaction, and perceived partner responsiveness on the effects of the above factors on the appraisal of vaginal pressure and sexual arousal responses. Significant interaction effects involving these quantitative variables were decomposed using simple slope analyses, testing effects at low (Mean − 1 SD) and high (Mean + 1 SD) values of the moderator variable. For exploratory reasons, we also examined whether men’s estimations of sexual arousal and painful pressure in their female partner were related to their own level of sexual arousal.

## Results

### Correlations Between Men and Women’s Reports

The correlational analyses between men and women’s responses revealed that their own reports of sexual arousal were not significantly related, neither in the sexual film condition, − .08 < all *r*’s < .03, all *p*’s > .10, nor in the neutral film conditions, − .11 < all *r*’s < .01, all *p*’s > .10. In addition, we found that women’s reports of painful vaginal pressure and men’s estimations of painful pressure were positively related in the conditions where no vaginal pressure was induced, *r*(42) = .45 *p* = .004 (neutral film) and *r*(42) = .41 *p* = .009 (sex film), but no significant correlation was found when vaginal pressure was actually induced, *r*(42) = .26, *p* = .106 (neutral film) and *r*(42) = .10, *p* = .560 (sexual film). When examining the correlation between men’s estimations of sexual arousal in women and their own level of sexual arousal, a positive correlation was found in the sexual film conditions with, *r*(42) = .53, *p* < .001, and without vaginal pressure induction, *r*(42) = .41, *p* = .010, but not in the neutral film conditions, − .13 < all *r*’s < .02, all *p*’s > .10. Furthermore, men’s estimations of painful pressure in their female partner were not significantly related to their own level of sexual arousal, neither in the sexual film condition, 0.3 < all *r*’s < .11, *p*’s > .10, nor in the neutral film conditions, 0.2 < all *r*’s < .21, all *p*’s > .10.

### Painful Pressure Ratings

On the aggregate level, incongruence implies a gender effect on the mean ratings of painful pressure and sexual arousal as reported by the women and estimated by the men. Table 1 shows the means and standard deviations of the outcome variables as a function of experimental condition. First, we studied the gender effect on painful pressure ratings as a function of pressure induction and film type, and we found a significant three-way interaction at *α* = .05; *F*(1, 40) = 4.54, *p* = .039. Follow-up analyses showed that gender interacted with film type, only in the condition with vaginal pressure, *F*(1, 40) = 9.78, *p* = .003. On average, men overestimated women’s level of painful pressure when watching a sexual film, *t*(40) = −2.19, *p *= .035, but not when watching a neutral film, *t*(40) = 1.14, *p* = .261.

**Table 1:** Means and SDs of the outcome variables as a function of experimental condition

	Neutral + pressure		Neutral − pressure		Sex + pressure		Sex − pressure	
	*M*	SD	*M*	SD	*M*	SD	*M*	SD
Sexual arousal W	2.23	1.66	1.63	1.15	3.58	1.87	3.28	1.62
Sexual arousal M	1.13	0.33	1.10	0.30	2.80	1.44	2.93	1.35
Painful pressure W	2.45	1.60	1.15	0.70	1.93	1.29	1.20	0.85
Men’s estimation of painful pressure in W	1.30	0.72	1.13	0.33	2.95	1.43	2.95	1.22
Men’s estimation of sexual arousal in W	2.13	1.34	1.63	1.00	2.55	1.40	1.93	1.23

All scores range on a scale from 1 to 7, with higher scores indicating higher sexual arousal and painful pressure (estimations)

*W* women, *M* men

Before examining the moderating role of relationship satisfaction and perceived partner responsiveness, we first explored potential gender differences in the variables of interest. As shown in Table 1, men and women did not significantly differ in their level of relationship satisfaction and perceived partner responsiveness. Both moderator variables were interrelated in men, *r *(42) = −.78, *p* < .001 and in women, *r *(42) = −.60, *p* < .001. We also found significant correlations across the perceived partner responsiveness and the relationship dissatisfaction scores of men and women, *r*(42) = .36, *p* = .020 and *r*(42) = .55, *p* < .001, respectively (Table 2).

**Table 2:** Means and SD of the moderator variables

	Women		Men		
	*M*	SD	*M*	SD	*t*
Relationship dissatisfaction	0.86	0.57	0.82	0.64	0.47
Perceived partner responsiveness	7.78	.91	7.83	0.66	− 0.38

Relationship satisfaction scores range on a scale from 0 to 8, with higher scores indicating relationship dissatisfaction

Perceived partner responsiveness scores range on a scale from 1 to 9

**Fig. 1:**
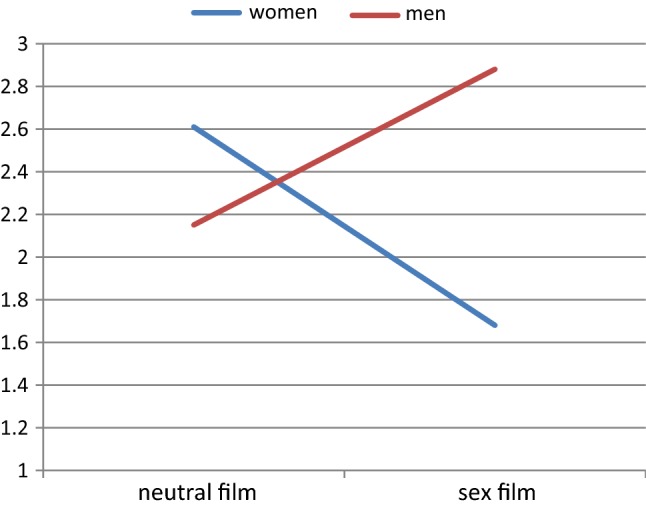
Interaction between gender and film type on ratings of painful pressure of women and estimation by men at high levels of male relationship dissatisfaction when vaginal pressure was induced

When considering the moderating impact of relationship satisfaction on painful pressure reports, we found that the previously described three-way interaction was qualified by a significant four-way interaction between male relationship satisfaction, gender, film type, and pressure, *F*(1, 40) = 7.31, *p* = .010. When decomposing this interaction, we found that, at high values of male relationship dissatisfaction, gender interacted with film type only in the pressure condition, *F*(1, 40) = 17.23, *p* < .001. Figure 1 shows that, at high levels of male relationship dissatisfaction, men on average overestimated the level of painful pressure in their female partner when watching a sexual film, *t*(40) = −2.20, *p* = .042, but not when watching a neutral film, *t*(40) = 1.37, *p* = .189.

**Fig. 2:**
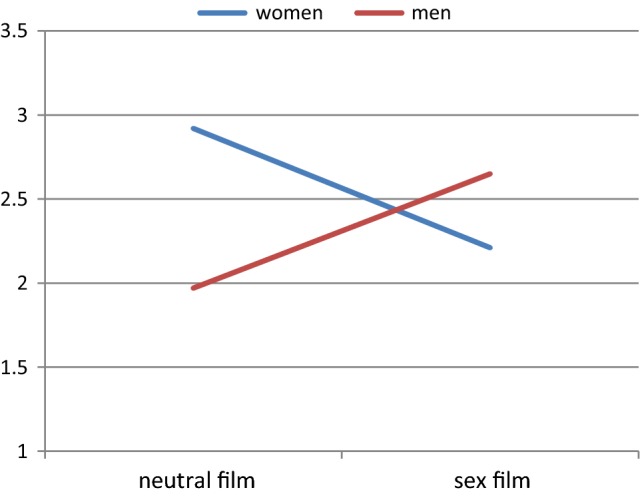
Interaction between gender and film type on ratings of painful pressure of women and estimation by men at low levels of female perceived partner responsiveness when vaginal pressure was induced

Also perceived partner responsiveness as reported by the women was found to have a moderating effect, revealing a marginally significant four-way interaction between perceived partner responsiveness, gender, pressure, and film type, *F*(1, 40) = 3.95, *p* = .054. When decomposing this interaction, we found that, at low values of perceived male partner responsiveness, gender interacted with film type only in the pressure condition, *F*(1, 40) = 10.13, *p* = .003. Figure 2 shows that, when women perceived their male partner as less responsive, men on average underestimated the level of painful pressure in their female partner when watching a neutral film, *t*(40) = 2.26, *p* = .037, but not when watching a sexual film, *t*(40) = −.86, *p* = .404.

### Sexual Arousal Ratings

**Fig. 3:**
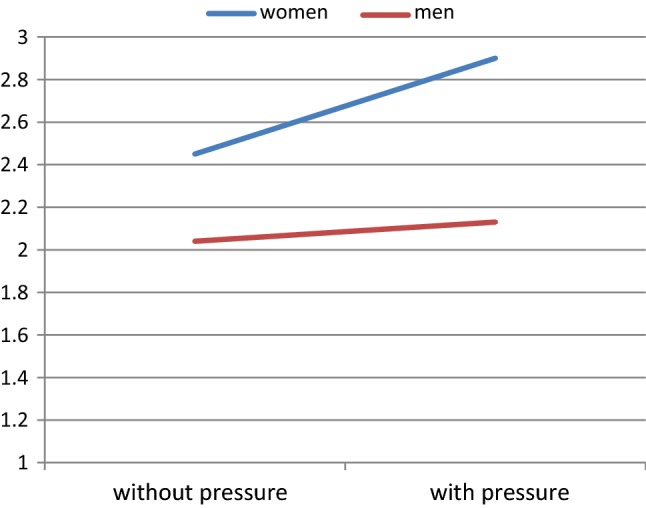
Interaction between gender and pressure on ratings of sexual arousal of women and estimation by men

Regarding the level of congruence in mean sexual arousal scores, no significant interaction was found between gender, pressure, and film type, *F*(1, 40) = .20, *p* = .660, although we did find a significant two-way interaction between gender and pressure, *F*(1, 40) = 5.27, *p* = .027. Figure 3 shows that the discrepancy between men’s estimated and women’s actual level of sexual arousal was larger when vaginal pressure was induced, with men underestimating the level of sexual arousal in their female partner.

**Fig. 4:**
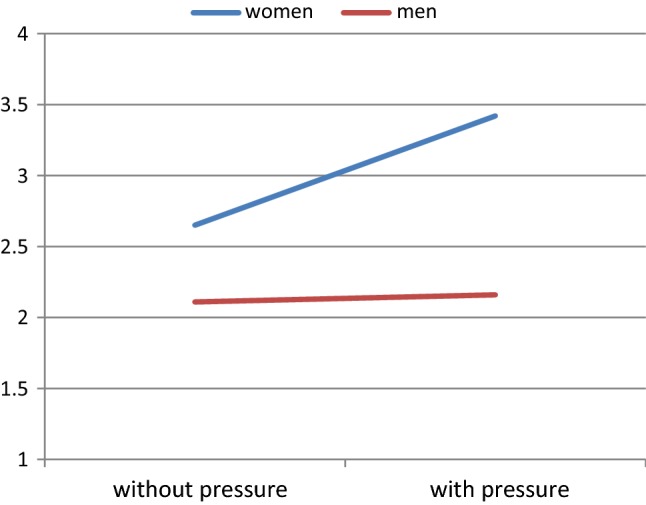
Interaction between gender and pressure on ratings of sexual arousal of women and estimation by men at high levels of female relationship satisfaction

Relationship satisfaction in women was found to moderate this effect, revealing a significant three-way interaction between gender, pressure, and relationship satisfaction, *F*(1, 40) = 4.54, *p* = .040. Follow-up analyses showed that, when women were highly satisfied with their relationship, the discrepancy between men’s estimation and women’s actual level of sexual arousal was larger when vaginal pressure was induced (Fig. 4). Perceived partner responsiveness, as reported by women and by men, showed no moderating effects on sexual arousal responses, all *F*’s < 1.50, *p*’s > .10.

### Sexual Arousal Effects on Pain Estimations

Because it is unclear whether women who watched the entire film clip are sexually more aroused than women who ended the procedure because of unpleasant sensations, higher pressure thresholds may indicate higher sexual arousal which may, in turn, affect pain estimations. To explore this possibility, we first performed correlations between sexual arousal and pain ratings in women and then examined the moderating influence of women’s level of sexual arousal (for each of the 4 conditions) on pain estimations. Women’s sexual arousal ratings were not significantly related to their pain appraisal scores, − .22 < all *r*’s > −.03, and women’s sexual arousal scores did not moderate the effects of pressure and film type on pain estimations, all *F*’s < 1.24, all *p*’s > .10.

## Discussion

The present study examined the level of congruence between partners’ ratings of pain and sexual arousal in response to vaginal pressure sensations, thereby inferring the men’s ability to estimate the women’s experiences. Because the relational climate has shown to influence pain estimations (Hadjistavropoulos et al., 2011; Rosen et al., 2015), we also considered the moderating role of intimacy-related variables such as perceived partner responsiveness and relationship satisfaction. In general, we found higher disagreement in pain ratings when vaginal pressure was induced in the context of sexual stimulation, with men overestimating the level of pain in women. Also sexual arousal ratings diverged between partners, with men underestimating the women’s level of sexual arousal during the induction of vaginal pressure, regardless of whether they were watching a sexual or neutral film. Importantly, the level of congruence between actual and estimated ratings of pain and sexual arousal depended on how relationally satisfied men and women were and how validated and supported women felt by their partner. This study is the first to investigate dyadic associations between partners in response to experimentally induced vaginal sensations in a controlled laboratory design. It thereby makes an important contribution to the growing literature on the social determinants of sexual and pain experiences.

### Congruence in Pain Appraisals

The finding that reports of pain and sexual arousal did not converge between partners was to be expected. Perfect agreement is unlikely when considering that the perceiver’s estimate inevitably contains systematic bias, even when responses are measured in real time (Kappesser & Williams, 2010). Previous research has revealed, however, that pain estimations between partners do covary (Cano et al., 2005; Leonard et al., 2013) and that men are fairly accurate in estimating changes in women’s pain sensations as measured using an 8-week daily diary (Rosen et al., 2015). On the other hand, this diary study also showed that, when considering the average pain ratings across days, men’s pain estimations did diverge from women’s actual pain reports. Men generally underestimated the pain of their partner, which contradicts our finding that men overestimated the women’s pain during the induction of vaginal pressure. One possible explanation for this divergence in findings is that previous work on pain estimations relied on a clinical sample of women suffering from genital pain, while the present study used an experimental procedure to elicit vaginal sensations in a sample of healthy volunteers. This resulted in fairly low pain appraisals during the experimental manipulation, which implies that we need be cautious when drawing conclusions about genital pain based on the current results. In a clinical sample in which the male partner is repeatedly confronted with genital pain, it makes sense that men will underestimate rather than overestimate the pain during sex because they may have learned to focus on the positive aspects of the sexual interaction and therefore dismiss pain signals to protect their own sexual enjoyment and integrity (Rosen et al., 2015).

The fact that the male partners in our study perceived, on average, more pain than the women were actually experiencing could thus be attributed to the current study design. We did not measure pain reports in response to a real sexual interaction; men simply observed while their partner underwent the experimental procedure. Because the men did not cause the pain themselves and were not involved in a sexual interaction with their partner, they may have been less motivated to accurately perceive, if any, pain signals of the women. Although the pain communication model would predict that women will exaggerate their pain expressions to elicit support from their partner (Hadjistavropoulos et al., 2011), it is possible that, during a real sexual encounter, women will inhibit and hide their pain because they are primarily focused on pleasing the partner (Ayling & Ussher, 2008; Elmerstig, Wijma, & Bertero, 2008). In the current study, women may have been less inclined to regulate their emotional expressions because the (threat of) pain was experimentally induced and thus external to the couple. Unfortunately, we did not measure the facial expressions of women which prevented us from exploring whether men’s pain estimations covaried with the women’s (non)verbal pain behavior. Note that our findings do fit with other research showing that caregivers tend to overperceive pain severity when observing another in pain (Clipp & George, 1992; Miaskowski, Zimmer, Barrett, Dibble, & Wallhagen, 1997).

Interestingly, men overestimated their partner’s pain only when watching a sexual film and not during a neutral film. One way to explain this finding is that men may have been so much focused on their own sexual arousal when watching a sexual film that they were less attentive to the actual signals of their partner, thereby providing inaccurate estimates that are driven by social desirability or overprotection biases. In this context, it is remarkable that we did not find a significant correlation between men’s level of sexual arousal and perceived level of pain in their partner. This was unexpected because we assumed that observing pain would elicit distress in men, which would then interfere with their subjective sexual arousal. Again, the fact that the male partner was not actively involved in the vaginal stimulation may have lowered his motivation to accurately estimate the women’s pain, thereby feeling less affected by the experimental procedure that was performed on their partner. Active participation of the men could be obtained, for example, by allowing them to control the amount of vaginal pressure to be induced. Being a co-actor instead of an observer may elicit different emotional and behavioral responses that better reflect the interpersonal dynamics involved in pain during sex. Another possible explanation is that women did not experience the vaginal pressure as painful when induced in the context of sexual stimulation. Lower pain scores in women increase the chance of obtaining disagreement. Previous research using the VPI did indeed show that women ascribe a more pleasant meaning to the vaginal pressure when presented in a sexual compared to a neutral context (Dewitte et al., 2018a, 2018b; Melles et al., 2017). Although the general pattern of correlations showed only significant associations between sexual arousal and pleasant pressure, but not painful pressure, the results of our experimental manipulation clearly showed that the experience of vaginal pressure as pleasant or painful covaries with women’s level of sexual arousal in response to sexual stimulation.

### Congruence in Sexual Arousal

When turning to the results on sexual arousal, we found that men underestimated the level of sexual arousal their partner was actually reporting. Although men are generally found to display a sexual overperception bias (Haselton & Buss, 2000; Henningsen & Henningsen, 2010), research has shown that, in established relationships, men tend to perceive lower sexual interest in women’s sexual behavior than actually exists. This underperception bias would serve relationship dynamics as it protects men from feeling sexually rejected and increases women’s feeling of satisfaction and commitment (Muise et al., 2016). Another explanation for men’s perception bias is that women show fewer manifest signals of sexual arousal because they are socialized to inhibit their expressions of sexual desire.

### The Important Role of Relationship Intimacy

In line with pain communication—and intimacy models of pain (Cano & Williams, 2010; Hadjistavropoulos et al., 2011), we found that the relational environment makes an important contribution to the level of agreement between partners’ reports of pain and thus, by inference, the men’s ability to estimate the women’s pain. With regard to pain estimations, we found that the less satisfied men were with their relationship and the more they were perceived as unresponsive by their partner, the more men overestimated the pain women were actually experiencing when vaginal pressure was induced. On the one hand, men may be more likely to interpret the vaginal pressure as a possible threat when evaluating the relationship as distressing. On the other hand, a negative relational climate may lower men’s motivation to understand the woman’s pain experience, making them less attentive to pain-related cues and thus less accurate to estimate their pain (Rosen et al., 2015). That is, a stronger focus on feeling dissatisfied with the relationship may detract the partner from attuning to the women’s signals of pain because he pursues self-oriented goals that aim at diminishing his own level of distress (Vervoort & Trost, 2017). Such self-focus may result in empathic failures and create an invalidating emotional climate that lowers the couple’s confidence to cope with the threat of pain together (Dagan et al., 2014). In this context, it is worth noting that being able to estimate the women’s inner feelings and sensations is a key ingredient of empathic responding, which then contributes to the women feeling supported and understood (Goubert et al., 2005, 2013; Laurenceau et al., 1998; Laurenceau, Barrett, & Rovine, 2005).

A large theoretical and empirical literature has identified relational intimacy as a protective factor in the face of adversity, leading to a better prognosis and adaptation to any health condition (Blasi, Harkness, Ernst, Georgiou, & Kleijnen, 2001; Uchino, Cacioppo, & Kiecolt-Glaser, 1996). Our finding that pain reports varied as function of perceived partner responsiveness and relationship satisfaction, which are both key determinants of relational intimacy (Reis et al., 2004), further supports the beneficial role of intimacy in tuning partners’ responses. Feeling validated and understood by the partner and experiencing a general sense of contentment with the relationship may encourage both partners to express and respond to pain in an unbiased way (Zaki, Bolger, & Ochsner, 2008). Note that the link between pain reports and relationship intimacy is likely to operate in a bidirectional way. That is, partners whose self-reported and estimated ratings of pain show better agreement will enjoy greater satisfaction with their relationship, in the same way as a more satisfying relationship context will make partners better attuned toward each other.

Given that women are generally perceived as more interpersonally oriented than men (Basson, 2000; Wood, 1996), it is surprising that pain reports were not influenced by women’s relationship satisfaction. It was specifically how men felt about their relationship and how they were perceived by the women that moderated the level of agreement between partners’ pain responses. Interestingly, women’s level of relationship satisfaction did moderate the effects on sexual arousal responding. The level of disagreement between partners’ ratings of sexual arousal increased when women reported greater satisfaction with their relationship. Hence, assuming that a satisfying relationship context fuels the women’s motivation to experience sexual desire (Basson, 2000; Meana, 2010), the sexual underperception of men could, in part, be accounted for by women’s high levels of sexual arousal in the context of a fulfilling relationship.

As a final observation, we want to draw attention to the complementarity between men’s estimates of pain and sexual arousal. It makes sense that men will estimate less sexual arousal in their partner when also estimating higher level of pain. This is clinically relevant because low levels of sexual arousal increase the risk of (if not cause) genital pain (Spano & Lamont, 1975; ter Kuile et al., 2010), which implies that the male partner should refrain from penetrative sex when perceiving low sexual arousal in the woman.

### Limitations

This is one of the first experimental laboratory studies to investigate the level of agreement between partners. There are, however, a few issues that need to be addressed in future work. First, our results yield only correlational data, so we cannot draw conclusions on the causal role of partner incongruence in shaping pain and negative sexual experiences. Because we did not measure relationship satisfaction and perceived partner responsiveness in real time during the laboratory procedure but as general indicators of relational intimacy over the past 6 months, we considered these as moderator variables and not as outcome variables. Furthermore, the results on relationship dissatisfaction should be interpreted with caution because our sample consisted of relatively satisfied couples. It is relevant to study the level of agreement between partners in couples that vary more strongly in their level of relationship and sexual functioning. In addition, despite a mainly within-subjects design, our sample size was quite small, which reduced the power (35% power to find a small effect of 0.125).

Another limitation is that our laboratory design did not reflect a naturalistic representation of interactive sexual activity. The fact that the male partners only passively observed the experimental procedure may have affected their own and estimated emotional responses. Furthermore, we did not systematically monitor possible interactions between partners, so we don’t know whether partners exchanged non-verbal signs and interactions.

Unfortunately, a technical failure prevented us from determining the pain thresholds, i.e., the time taken to reach the threshold of unpleasant vaginal pressure from the start of the pressure to the stop of the pressure/film. We were thus not able to examine whether men’s tendency to overestimate pain varies as a function of the vaginal pressure threshold. Future research should focus on the pressure threshold to understand whether pain estimations and congruence levels differ when women persist versus break off the 5 min of vaginal pressure.

Finally, only indirect conclusions can be made on partner incongruence and pain estimations in the context of genital pain because we did not use a clinical sample of women with genital pain and we did not focus on pain sensations as women were instructed to terminate the vaginal pressure when it started to feel unpleasant rather than painful. This was clearly indicated in the pain appraisals we obtained, which were generally low. It is therefore important to replicate this study in a clinical sample. Note that genital pain is a heterogeneous condition (Dewitte et al., 2018a) and that the design of this study focused mainly on pain during introitus and can thus not be generalized to all types of genital pain (e.g., deep dyspareunia). Furthermore, the VPI simulates only the feeling of initial pressure upon penetration but does not include dynamic thrusting which is characteristic of penetrative sex.

### Concluding Remarks

Our findings highlight the importance of examining the level of (dis)agreement between both partners’ responses when studying sexual dynamics in the context of potentially painful experiences. Being able to estimate how the other partner is feeling allows one to adjust and tune behavioral and emotional responses. Inaccurate perceptions, on the other hand, can lead to feelings of invalidation, dissatisfaction, and distress (Cano et al., 2005; Rosen et al., 2015). Within such dyadic perspective, it is relevant to investigate whether and in what way partner (in)congruence leads to tangible relational and sexual costs or benefits.

## Footnotes

1 We will use the term genital pain throughout this article because this aligns with the heterogenous nature of genital pain and covers a broad spectrum of pain disorders (Dewitte, Borg, & Lowenstein, 2018a; Dewitte, Schepers, & Melles, 2018b). Given that the present study does not include a clinical sample, we do not mention the DSM-5 diagnosis of Genito-Pelvic Pain/Penetration disorder to avoid any possible confusion.

2 Note that perfect agreement implies a perfect association, as well as an equal mean score.
